# Social App to Improve Warfarin Therapy in Post-MHVR Chinese Patients: A Randomized Controlled Trial

**DOI:** 10.1155/2023/2342111

**Published:** 2023-01-14

**Authors:** Litao Zhang, Sha Li, Zishuo Li, Dan Yu, Haiyan Wu, Bing Hua, Li Xie, Xia Yuan, Yun Li, Zhenlu Zhang, Yanli Long

**Affiliations:** ^1^Clinical Laboratory, Wuhan Asia General Hospital Affiliated to Wuhan University of Science and Technology, Wuhan, China 430056; ^2^Anticoagulation Clinic, Wuhan Asia Heart Hospital, Wuhan, China 430022; ^3^Cardiac Surgery, Wuhan Asia Heart Hospital, Wuhan, China 430022; ^4^Administrative Office, Wuhan Asia Heart Hospital, Wuhan, China 430022; ^5^Nursing Department, Wuhan Asia Heart Hospital, Wuhan, China 430022; ^6^Clinical Laboratory, Wuhan Asia Heart Hospital, Wuhan, China 430022

## Abstract

**Background:**

Poor anticoagulation quality was a major problem among warfarin-treated patients, which called for innovative and effective methods to improve it.

**Objective:**

To investigate whether social app could be used to reduce warfarin-associated adverse events among post-MHVR Chinese patients.

**Method:**

735 warfarin-treated patients (aged 50.8 ± 9.6 years, 59.9% female) were enrolled and randomized to a social app care group (warfarin therapy was guided by experienced clinicians via a social app) or a routine care group (warfarin therapy was managed through traditional in-office visits) at a 1 : 1 ratio. Ending points (bleeding and thrombotic events) were recorded during an 18-month follow-up period.

**Results:**

A total of 718 patients were included in analysis. 57 of them suffered warfarin-associated adverse events, including 30 major bleedings and 27 thrombotic events. The time in the therapeutic range (TTR, Rosendaal method) in the social app group was 71.5%, which was significantly better than 52.6% in the routine care group (difference: 18.8%, 95% CI: 16.8-20.8). Compared with the patients from the social app group, patients under routine care experienced more bleeding (hazard ratio (HR): 2.31, 95% CI: 1.13-4.72). The social app care group had lower variation (0.55 vs. 0.70) in the international normalized ratio (INR) values and fewer incidents of extremely high INR (e.g., INR > 5.0, 0.87% vs. 3.42%) than the routine care group.

**Conclusions:**

Social app management could significantly improve warfarin control and was associated with a reduction in bleeding risk. This trial was registered with NCT03264937.

## 1. Introduction

Warfarin, a vitamin K antagonist (VKA), has been used to prevent thrombosis for decades. Currently, VKAs are still the only anticoagulants provided to patients who have undergone mechanical heart valve replacement (MHVR) [1, 2], and are still widely used for patients with atrial fibrillation (AF) or other clinical conditions [3], although the use of direct oral anticoagulants is widespread [4]. Thus, the accumulated number of populations that uses VKAs is incredibly high [5]. Generally, the international normalized ratio (INR) is routinely monitored to ensure the efficacy and safety of VKAs. Anticoagulation control plays a key role during long-term VKA therapy and is usually measured by time in the therapeutic range (TTR, %). Previous studies have demonstrated that increasing of TTR levels are associated with a reduction of anticoagulation-related adverse events [6, 7].

Previous studies concluded that anticoagulation clinics, online management, and self-management could significantly improve the effects of warfarin therapy in Western countries [8–10]; however, while these strategies are common in Western countries, these approaches are not common in China due to differences in patient education, culture, lifestyles, and medical models. Currently, most warfarin-treated patients in China still need to visit the outpatient departments of their central or local hospitals, which are usually located downtown, to receive anticoagulation management. This routine care usually requires large amounts of time, traffics, or costs, which might reduce patient compliance. It has been reported that routine care resulted in poor anticoagulation quality among Chinese patients (TTR, 38.8%-51.7%) [6, 11], that is at a much lower level than the recommended level (TTR ≥ 70%) [12]. Even with the SAMe-TT2R2 evaluation, a clinical prediction tool to predict the TTR in AF patients [13], Chinese (nonwhite) patients will score at least 2 points, which is considered predictive of a low TTR [14, 15]. Together, these situations indicate that improving anticoagulation quality is an urgent issue for Chinese patients. Notably, the measures employed to improve anticoagulation quality should be suited to specific conditions. For example, in a cluster-randomized trial, Victor et al. found that among 319 non-Hispanic black male barbershop patrons with uncontrolled hypertension, health promotion by barbers coupled with medication management in barbershops by specialty-trained pharmacists resulted in larger blood-pressure reductions than reductions with standard management afforded by primary care practices [16]. Since the factors that affect TTR differ greatly among different regions and populations [17], therefore, we need to determine these factors and develop innovative measures to improve TTR according to local conditions and population characteristics. We found that almost every Chinese adult frequently uses social network services. It would be very interesting that social media have a similar influence on Chinese patients.

Social apps have changed and continue to change the world by making communication easier and more accessible than ever before. People can use social apps to contact each other at any time and in any location via pictures, words, voice recordings, video, etc. WeChat (Tencent, Shenzhen, China) has nearly 1.1 billion active users and is the most popular and most frequently used social media app in China [18]. Almost every Chinese adult has a WeChat account. Recently, WeChat has become an open platform on which different organizations can set up various WeChat miniprogrammes, which are “subapplications” within the WeChat ecosystem that enables one-to-one or one-to-many features to WeChat users in professional vertical fields, such as e-commerce, task management, and customer service [19]. However, there have been no reports on whether this social app can be used to manage warfarin therapy.

Therefore, we set up an approved WeChat miniprogramme (Warfarin Helper) and conducted the SMART (Social Media to improve Warfarin Therapy) trial to assess whether health care with a social app could be used to improve warfarin control and reduce warfarin-associated adverse events among post-MHVR Chinese patients.

## 2. Patients/Methods

### 2.1. Trial Design and Oversight

The study was a prospective, open label, and randomized controlled trial conducted in Wuhan Asia Heart Hospital in China. The study compared the primary and secondary outcomes between patients receiving warfarin therapy who received social app management and those who received routine management. The protocol was designed by a multidisciplinary team that included doctors, biomedical scientists, pharmacists, and nurses. No changes were made to the protocol design, eligibility criteria, or outcome evaluations once the trial started. The protocol was conducted in accordance with the code of ethics of the World Medical Association (Declaration of Helsinki) for experiments involving humans. Ethical approval for this study was obtained from the Wuhan Asia Heart Hospital ethical committee (2017-YXKY-B012). The full trial protocol was also registered at the registry of clinical trials (Clinical Trials.gov; NCT03264937) before commencing recruitment. All enrolled patients provided written informed consent.

### 2.2. Participants and Randomization

Trial enrolment was completed between September 2017 and February 2018. Patients were eligible for inclusion if they were aged from 18 to 65 years and had undergone implantation of a mechanical valve in the aortic or mitral position or both. Patients who had suffered from recent (within 30 days) ischaemic stroke, cerebral haemorrhage, or other serious diseases and female patients who were planning to be pregnant were excluded.

Eligible patients were invited to participate in the study when they joined our warfarin education course. We used a random number generator to randomize the patients after enrolment at a ratio of 1 : 1. No patients were involved in the design of our study or the development of research questions and outcome measures. None of the patients were involved in patient recruitment or in conducting the study. No patients took part in the assessment of the intervention. There were no plans to involve patients in the dissemination of the study results.

### 2.3. Intervention and Follow-Up

Patients began taking warfarin after undergoing valve surgery. Based on a previous study and clinical practice in China [7, 20], we employed an INR target of 2.0 (1.5-2.5) for aortic valve replacement (AVR) patients and 2.3 (1.8-2.8) for mitral valve replacement (MVR) or double valve replacement (DVR) patients, both of which were lower than the recommendations stated in the established guidelines. Low anticoagulation intensity has been well demonstrated as an optimal strategy for Chinese patients in our previous study [20]. The doctors were able to individually adjust the target range for certain patients. All patients were asked to monitor INR once per month at least and were followed up for 12-18 months after randomization unless the first primary clinical outcome occurred.

**Figure 1:**
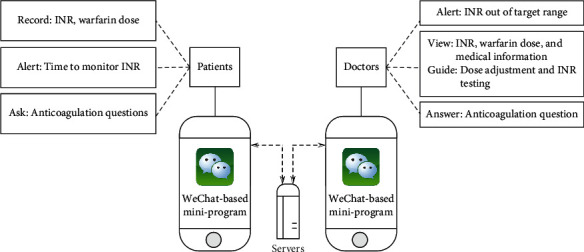
The pattern diagram of warfarin management via WeChat-based miniprogram. INR = international normalized ratio.

Intervention (social app care): In the social app group, patients were asked to register and use an approved WeChat miniprogramme named Warfarin Helper, which helped to manage their warfarin therapy. The miniprogramme mainly had the following four functions, as shown in Figure 1: (1) patients could use the miniprogramme to continuously record every INR result; (2) the miniprogramme could alert the patients to monitor their INR regularly and alert doctors if the patient's INR was out of the target range; (3) patients could ask questions or provide additional information by sending text messages or photos of the results one-to-one via the miniprogramme; and (4) doctors could use the miniprogramme to guide dose adjustments or answer questions one-to-one based on the individual's medical information and INR results. These patients monitored their INR results according to the investigators' advises via the miniprogramme, they could use self-testing instruments or do INR tests in their local hospitals (including our hospital).

Control (routine care): patients who were assigned to the routine care group received traditional warfarin management. Warfarin-treated patients were asked to independently visit their local hospitals to monitor their INRs and receive dose adjustments. These patients were asked to give or send us their INR reports when they visited our centre for rehabilitation examinations every three to six months.

The algorithm recommended that the dose be calculated on a weekly, rather than daily basis because the recommended dose changes were small and difficult to achieve with daily dosing [21]. The algorithms of dose adjustment in our study were as follows: the algorithm recommended no change, increase, or decrease in the weekly warfarin dose based only on the current INR, such as no change for INR in goal range, increase 5-15% of dosage for INR ≤ 1.50, and decrease 10-15% for INR 2.81 to 3.50. For INR 3.51 to 4.99, the recommendation was to hold the dose for two or three days and then reduce it by 10-15%. For INR ≥ 5.00, the dose was to be held until the INR was therapeutic and then decreased by 20% per week. The investigators were asked to use this algorithm individually and converted the weekly dose to a receptive daily dose for patients. Weekly INR monitoring was recommended for out-of-range INR values.

### 2.4. Privacy Protection and Data Security

The privacy policy was carefully reviewed and approved by the Ethical Committee. On basis of the Warfarin Helper, a miniprogramme based on WeChat platform, the medical information and anticoagulation data could be only viewed or accessed by the subject himself and the research team. Each participant read and agreed to our privacy policy before using the applet. The data obtained from the applet were stored in the hospital servers, which are protected and monitored by the hospital medical information committee.

### 2.5. Primary Clinical Outcomes

The primary clinical outcomes were major warfarin-related events, including thrombotic events or major bleedings. Specifically, a thrombotic event refers to valve thrombosis, transient ischaemic attack, ischaemic stroke, peripheral embolism, and myocardial infarction [22]. The occurrence of a major bleeding was defined according to the International Society on Thrombosis and Hemostasis [23], which involved cerebral haemorrhage, gastrointestinal bleeding, and other internal or external bleeding that led to death, hospitalization, or permanent injury (e.g., vision loss) or necessitated transfusion.

### 2.6. The Secondary Endpoints

The secondary endpoints included TTR, INR variation, and time periods of extremely high INR. TTR was calculated according to Rosendaal et al. [24]. If a gap longer than 90 days between two INR values occurred, then the period was excluded from the patient's TTR calculation. The INR variation was evaluated as the standard deviation of the INRs [25]. The times of extremely high INR were mainly referred to times of INR > 5.0 and INR > 12.0 according to a previous study [26] and the statement of critical values in our hospital. In addition, the percentage of individual TTRs above 70% was also calculated to assess anticoagulation quality. All INR results were retrieved from a database where the INR values and exact treatment times were registered. The INR results in the social app group were recorded by the participants and validated by our team; for the control group, the INR records were obtained from the patients' coagulation reports or anticoagulation tables.

### 2.7. Sample Size

We carried out the study with two groups at a ratio of 1 : 1 and determined the sample size by using an online estimation tool (http://powerandsamplesize.com/Calculators). The incidence of all warfarin-related events was reported as approximately 12.2% in post-MHVR patients with TTR < 70% [7]. A previous model reported that a 10% increase in TTR could independently predict a 20% reduction in the rate of composite clinical outcomes among patients on warfarin [21]. Accordingly, if we expected to observe a 30% increase in TTR compared to that of the control group, at least 608 patients in total would be required to observe a relative reduction of 60% in the rate of primary events with 90% power and a two-sided *α* = 0.05. Thus, after adjusting for potential dropouts, we recruited more than 700 patients for our study, with over 350 patients in each group.

### 2.8. Statistical Analysis

Continuous variables were expressed as the mean ± SD or median. Categorical variables were presented as absolute numbers or percentages. The differences between groups were assessed by *t*-test or Mann–Whitney *U* test for continuous variables and by chi-square test or Fisher exact test for categorical variables. The primary outcomes were visualized by the Kaplan-Meier event-free curve. The hazard ratio (HR) and 95% confidential interval (95% CI) were compared by using log-rank tests. A logistic regression analysis was performed to identify the determinants of high TTR levels. A *P* < 0.05 was considered statistically significant. The data was analyzed with GraphPad version 5.00 (GraphPad Software, California USA) and Medcalc version 16.2 (Medcalc Statistical Software, Ostend, Belgium).

## 3. Results

### 3.1. Patients and Follow-Up

**Figure 2:**
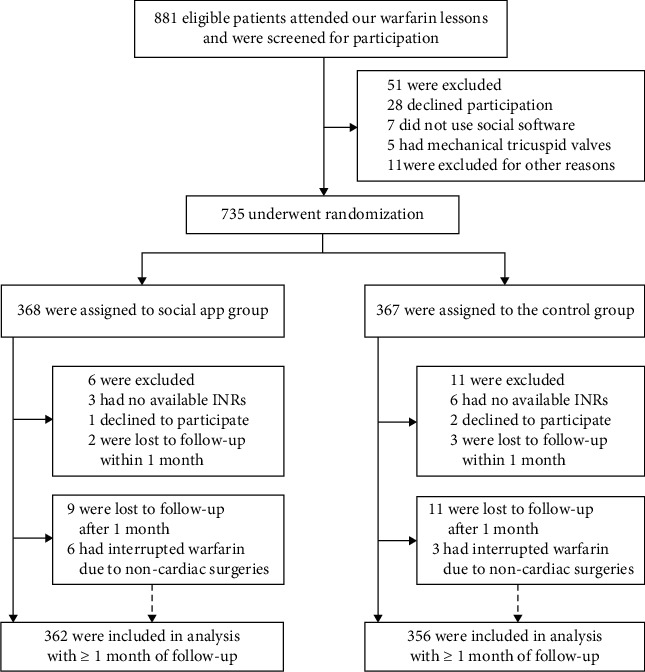
Enrolment and flowchart of the study.

The study occurred from September 2017 through March 2019.  Figure 2 shows a diagram of the entire study population. Of the 881 patients who joined our warfarin education lessons and were screened, 735 patients including 544 new warfarin users and 191 experienced warfarin users, were randomly assigned to a group to either receive social app management (*n* = 368, social app group) or receive routine care management (*n* = 367, routine care group).

All study participants were followed up for at least 12 months; the mean duration of follow-up was 491 days. During the follow-up period, 20 patients were excluded from the study. Three declined to participate in the study, and 9 were excluded because of failure to obtain available INR results. A total of 25 patients were lost to follow-up; of these patients, 5 were excluded because they were followed up for less than one month after randomization, but the other 20 were included in the analysis. Another 11 patients interrupted their warfarin treatment for noncardiac operations, such as dental operations and hernia operations, but all of these patients resumed warfarin therapy afterwards. Overall, a total of 718 patients completed the study and were included in the analysis.

The baseline characteristics of the patients are shown in Table 1. The mean age of the study population as a whole was 50.8 ± 9.6 years (19 to 65 years), and 59.9% of the patients were female. Compared with patients assigned to the routine care group, more patients in the social app group were aged below 60 years (*P* = 0.04). Patients from the social app group underwent more INR tests (*P* < 0.001) and used more self-testing (*P* < 0.001) than those from the routine care group. The average of INRs was significantly lower for the social app group than for the routine care group (*P* = 0.005). No significant differences were observed between the two groups in body mass index, valve position, smoker, medical histories, drug combinations, and other baseline characteristics, respectively (Table 1).

### 3.2. Primary Outcomes

**Figure 3:**
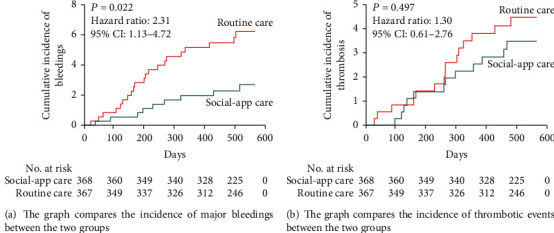
Kaplan-Meier analysis of the cumulative incidence of events.

The primary outcomes are listed in Table 2. In total, 57 events were observed during the follow-up period. Of the 30 patients who had major bleedings, 21 received routine care management. Among the 27 patients who experienced thrombotic events, 15 were in the routine care group. No deaths occurred during the whole follow-up period.  Figure 2 shows the Kaplan-Meier estimates of the time to the first primary event, which were based on up to 18 months of follow-up data. Compared with the social app group, the routine care group had substantially more major bleedings (*P* = 0.022 by log-rank test, Figure 3(a)), and the unadjusted HR in the social app group was 2.31 (95% CI: 1.13-4.72). After adjusting for age below 60 years, sex, average of INRs, and amiodarone use for major bleedings, the adjusted HR was 2.30 (95% CI: 1.04-5.69, Table 2). The incidence of thrombotic events was not significantly different between the two groups (adjusted HR: 1.17, 95% CI: 0.0.54-2.55, *P* = 0.685, Table 2; unadjusted HR: 1.30, 95% CI: 0.61-2.76, *P* = 0.497, Figure 3(b)).

### 3.3. The Secondary Endpoints

Table 2 summarizes the secondary endpoints in this study. During the study period, the mean TTR of the social app group was 71.5%, which was much higher than the 52.1% of the routine care group (absolute difference: 18.8%, 95% CI, 16.8 to 20.8; *P* < 0.001). The social app group had a higher percentage of patients with TTR ≥ 70% (58.0% vs. 8.15%, odds ratio: 15.9, 95% CI: 10.3-24.6, *P* < 0.001) and a lower INR variation (0.55 vs. 0.70, absolute difference: -0.15, 95% CI: -0.18-0.11, *P* < 0.001) than the routine care group.

Among a total of 9425 INR results in the social app group, there were 82 of them above 5.0, the incidence of INR values over 5.0 was much lower in the social app group than INR results in the routine care group (245/7169) (0.87% vs. 3.42%, *P* < 0.001, Table 2). In particular, only one INR > 12.0 result occurred in the social app group, and it happened in an elderly woman who took the wrong dose for two weeks. The incidence of INR values over 12.0 was also much lower in the social app group when compared with the routine care group (27/7169) (0.00% vs. 0.38%, *P* < 0.001, Table 2).

### 3.4. Determinants of High TTR

Table 3 shows the determinants of good TTR levels (≥70%) identified from a logistic regression analysis. The univariate analysis showed that a low number of complications, no amiodarone use, low average of INRs, self-testing, high frequency of monitoring, and social app care were significant determinants of a good TTR level. The multivariate analysis that included these 6 parameters revealed that 5 of these parameters, in addition to the frequency of monitoring, were independent predictors of good TTR levels.

## 4. Discussion

The results of our SMART trial indicate that social app care was associated with a reduced incidence of major bleedings during an average 491 days of follow-up. The anticoagulation quality, including TTR, INR variation, and time periods of extremely high INR, also significantly improved with social app care compared with routine care in post-MHVR patients receiving long-term warfarin therapy.

In the current study, social app care worked like a “real-time online anticoagulation clinic”. With the help of the social app, the warfarin treatments were not limited to long distances, which lowered traffic inconvenience and time costs to ensure a high frequency of monitoring and timely dose adjustments. Since the use of a social app removed barriers of communication between doctors and patients, the miniprogramme promoted the use of self-testing and has also been reported to be effective in improving warfarin control [9, 27]. Nonetheless, variation in physician experience was another factor that could affect warfarin control. Our experienced anticoagulation clinicians introduced many useful elements. For example, we used a weekly algorithm-consistent dosing strategy, which was based on experience from the RE-LY study, and was reported to independently increase TTR [21]. Finally, significant improvements in warfarin control resulted in a reduction in the number of major bleedings. Currently, many reports encourage doctors and patients to use “mobile health” to improve health care [28, 29]. Our study not only provides evidence for using social software to improve warfarin therapy, but also provides a new idea of using “mobile health” to optimize disease management. Particularly worth mentioning is that our social app care severed much more patients during the COVID-19 epidemic in Wuhan to manage those warfarin-treated patients without medical exposure, which might be helpful in reducing the risk of COVID-19 infection.

Previously, various efforts have been made to improve warfarin anticoagulation in China, including through pharmacist clinics and anticoagulant clinics [27]; however, these clinics for warfarin management in China were too few to sever a large numbers of warfarin-treated patients over the country. In contrast, our service could allow for rapid, extensive, and high-throughput expansion for patient management. Before creating the WeChat miniprogramme, we first tried two other ways of managing warfarin therapy. First, we tested an independent mobile app for Android devices. These apps were not based on social media, which meant that patients had to install and use the apps discretely. Unfortunately, warfarin-treated patients usually monitor their INR every couple of weeks and thus, the app was not used nearly as often as the social app; therefore, the Android app rapidly lost active users. In response, we then employed the most frequently used social app, which is both user-friendly and easy for patients to access during their daily life and can be installed regardless of which cell phone operating system the patient had. The results showed that the vast majority of patients were willing to use the app for a long time. Second, we tested a group chat. The group chat aimed to draw patients into a group for counselling and medication guidance. However, we found that this model had many disadvantages; for example, the group chat usually worked on one-to bases; thus, everyone in the group could see the medical information that was sent, indicating that this method failed to protect patient privacy. In addition, a crowded group chat usually led to the presence of a large amount of invalid information, which reduced the doctor response efficiency. Finally, the social app in the current study provides one-to-one service. This app might be the best approach to overcome all disadvantages.

The higher the TTR, the fewer the adverse events that occur. We observed a significant reduction in the number of major bleedings as the TTR increased, which was consistent with the results of the previous studies [6, 7, 21]. Even the “labile INR (TTR<60%)” has been introduced in the HAS-BLED score [30], which is a scoring system developed to assess the risk of bleeding in patients with AF taking VKAs. Compared to patients with high TTR levels, patients with low TTR levels were observed to have noticeably more high INR values, which have been well demonstrated to be associated with bleeding. This result might explain why the routine care group had higher bleeding rates than the social app group. Interestingly, multivariate analysis showed that five factors, including medical history, amiodarone use, average of INRs, self-testing, and social app care, were independently correlated with TTR levels. However, no significant correlation was observed between the SAMe-TT_2_R_2_ score and TTR levels in this study, which might be because our study included patients who underwent MHVR and not patients with AF. Our social app intervention had the biggest effects on TTR and may change the potential correlation between SAMe-TT_2_R_2_ score and TTR levels. These findings indicate that Chinese patients might need a modified prediction tool based on their own population to predict their TTR levels.

This study has several limitations. First, this study was a single-centre study, and selection bias may exist. Although we attempted to enrol all eligible subjects, 51 patients were excluded. In addition, the TTR of the control group was higher than that reported in previous literature [6, 17], indicating that the sample size of this study calculated based on a lower TTR which might be underestimated. Although we still observed significant differences in the number of major bleedings between the two groups, a larger sample size would reduce the statistical bias. Second, the dose adjustments were all based on the INR results provided by the patient. However, these results were tested with different analyzers or self-monitors, and the bias among different analysis systems is unknown. Third, the follow-up period was short. Post-MHVR patients need lifelong anticoagulation treatment, and long-term compliance has not been fully assessed. A long-term follow-up study might provide more evidence of the clinical benefits of our social app. Finally, although the number of patients does not nearly exceed what our team can handle, we expect that our service resources will be stretched if there is a significant increase in patient numbers. In future studies, we will explore the introduction of artificial-intelligence-based auto-management into our social app service (NCT03870581). In addition, whether social app management can be used in other patient populations, such as patients with AF, needs to be further evaluated in future studies.

## 5. Conclusions

Social app management could significantly improve warfarin control and was associated with a reduction in bleeding risk. Further studies are needed to confirm our findings and evaluate whether this idea can be used in the management of other diseases.

## Figures and Tables

**Table 1:** Baseline characteristics of the patients.

Characteristic	Social app group *N* = 362	Routine care group *N* = 356	*P* value
Age			
Mean age (yrs)	50.3 ± 9.8	51.3 ± 9.5	0.14
Age < 60 years (*n*, %)	298 (82.3)	271 (76.1)	0.04
Female (*n*, %)	218 (60.2)	212 (59.6)	0.85
Height (cm)	160 ± 7.8	160.2 ± 8.8	0.75
Weight (kg)	57.0 ± 10.1	57.2 ± 11.1	0.78
Body mass index	22.2 ± 3.4	22.2 ± 3.6	1.0
Valve position			
AVR (*n*, %)	74 (20.4)	74 (20.8)	0.91
MVR (*n*, %)	165 (45.6)	167 (46.9)	0.72
DVR (*n*, %)	123 (34.0)	115 (32.3)	0.63
Smoker (*n*, %)	102 (28.2)	99 (27.8)	0.91
Medical history			
Atrial fibrillation (*n*, %)	182 (50.3)	191 (53.7)	0.37
Diabetes (*n*, %)	14 (3.9)	18 (5.1)	0.47
Hypertension (*n*, %)	62 (17.1)	60 (16.9)	0.92
NYHA score ≥ II (*n*, %)	152 (42.0)	162 (45.5)	0.34
Stroke (*n*, %)	62 (17.1)	63 (17.7)	0.84
VTE (*n*, %)	2 (0.6)	1 (0.3)	0.57
Coronary artery disease (*n*, %)	22 (6.1)	32 (9.0)	0.14
Peripheral arterial disease (*n*, %)	1 (0.3)	3 (0.8)	0.37
Hepatic dysfunction (*n*, %)	14 (3.9)	19 (5.3)	0.38
Renal dysfunction (*n*, %)	7 (1.9)	9 (2.5)	0.65
SAMe-TT_2_R_2_ score	4.00 ± 0.74	3.97 ± 0.77	0.55
Drug combinations			
Aspirin (*n*, %)	28 (7.7)	40 (11.2)	0.11
P_2_Y_12_ inhibitor (*n*, %)	3 (0.8)	3 (0.8)	1.00
Amiodarone (*n*, %)	95 (26.2)	117 (32.9)	0.052
Average of INRs	2.40 ± 0.24	2.45 ± 0.31	0.005
Warfarin dose	3.35 ± 0.81	3.42 ± 0.77	0.24
Frequency of monitoring	26.0 ± 8.57	20.1 ± 6.47	<0.001
Self-testing (*n*, %)	113 (31.2)	27 (7.6)	<0.001
Follow-up (days)	496.9 ± 92.7	485.7 ± 110.9	0.14

The continuous variables in this table were normally distributed and expressed as mean ± SD. AVR: aortic valve replacement; MVR: mitral valve replacement; DVR: double valve replacement; NYHA: New York Heart Association; VTE: venous thromboembolism; INR: international normalized ratio. The SAMe-TT_2_R_2_ score considers the sex, age, medical history, treatment regimen, tobacco use, and race. An INR target of 2.0 (1.5-2.5) for AVR patients and 2.3 (1.8-2.8) for MVR or DVR patients. Average of INRs refers to the average of all the INR results during follow-up period.

**Table 2:** Primary and secondary outcomes.

End points	Routine care group *N* = 356	Social app group *N* = 362	Adjusted HR (95% CI)	*P* value
*Primary endpoints*				
Major bleedings (*n*, %)	21 (2.92)	9 (1.25)	2.30 (1.04-5.69)	0.0397
Thrombotic events (*n*, %)	15 (2.09)	12 (1.67)	1.17 (0.54-2.55)	0.685
*Secondary endpoints*			Difference or odds ratio (95% CI)	
Mean TTR (%)	52.6 ± 12.9	71.5 ± 14.6	-18.8 (-20.8 to -16.8)	<0.001
TTR ≥ 70% (%)	8.15 (29/356)	58.0 (212/362)	-15.9 (-24.6 to -10.3)	<0.001
INR variation	0.70 ± 0.24	0.55 ± 0.19	0.14 (-0.11-0.18)	<0.001
Extremely high INR				
INR > 5.0 (*n*, %, total)	245 (3.42, 7169)	82 (0.87, 9425)		<0.001
INR > 12.0 (*n*, %, total)	27 (0.38, 7169)	1 (0.00, 9425)		<0.001
*Event details*				
Cerebral haemorrhage (*n*)	3	2		
Gastrointestinal bleeding (*n*)	14	5		
Pulmonary haemorrhage (*n*)	1	0		
Other bleeding (*n*)	3	2		
Valve thrombosis (*n*)	2	1		
Stroke (*n*)	8	6		
Atrial thrombosis (*n*)	1	0		
Myocardial infarction (*n*)	2	3		
Peripheral embolism (*n*)	2	1		
Pulmonary embolism (*n*)	0	1		

TTR: time in therapeutic range; INR: international normalized ratio; HR: hazard ratio; 95% CI: 95% confidential interval.

**Table 3:** Determinants of high TTR levels (>70%) by logistic regression analysis.

	Total (*n* = 718)	Odds ratio (95% CI)	*P* value
Univariate analysis			
Age < 60	569		0.12
Female	430		0.45
Atrial fibrillation	373		0.19
Medical history^∗^		0.82 (0.69-0.98)	0.025
Amiodarone use	212	0.60 (0.42-0.86)	0.005
Smoker	201		0.19
SAME-TT_2_R_2_ score			0.45
Average of INRs		0.13 (0.07-0.24)	<0.001
Self-testing	140	3.73 (2.54-5.46)	<0.001
Frequency of monitoring		1.06 (1.03-1.07)	<0.001
Social app management	362	15.9 (10.3-24.6)	<0.001
Multivariate analysis			
Medical history^∗^		0.80 (0.65-0.98)	0.033
Amiodarone use		0.62 (0.40-0.97)	0.036
Mean INR		0.08 (0.03-0.19)	<0.001
Self-testing		1.87 (1.18-2.96)	0.008
Frequency of monitoring		1.00 (0.98-1.03)	0.48
Social app management		14.7 (9.07-24.0)	<0.001

INR: international normalized ratio.^∗^Medical history denotes number of the following conditions: hypertension, diabetes, coronary artery disease, peripheral arterial disease, heart failure, stroke, and pulmonary, hepatic, or renal disease.

## Data Availability

The .xls type data used to support the findings of this study are currently under embargo, while the research findings are commercialized. Requests for data, 12 months after publication of this article, will be considered by the corresponding author.

## Additional Points

*Bullet Points*. (i) High quality of anticoagulation control was associated with a reduction of warfarin-related outcomes. (ii) Anticoagulation quality urgently needs to be improved in Chinese patients receiving warfarin therapy. (iii) Social app could be used in the management of warfarin therapy in Chinese patients. (iv) Social app care could significantly increase anticoagulation control and reduce bleeding risk during warfarin therapy.
